# 

**Published:** 1872-04

**Authors:** 


☞ Should any of our Subscribers or Exchanges fail to get THE COMPANION regularly, they will confer a favor by notifying us of the fact.

☞ Our Mailing Clerk will faithfully forward THE COMPANION. Subscribers will please notify Post-masters that it will reach their offices, if not lost on the way, between the 15th and 20th of every month. Should it fail to come, after a reasonable time, notify the Editors, and it will be sent.

☞ We respectfully solicit Original Articles, Reports of Societies, Clinics, Home and Foreign Correspondents, News, etc., etc., of interest to medical readers.

☞ To insure publication, articles should be practical, brief as possible, and carefully written, on ONE side of the paper. Articles should be received by the 15th of one month to insure publication in the next. ☞ Friends, send in your articles. ☜

☞ All Subscribers and others will please write their names, post-offices, county and State *plainly*. ☞ *Remember, this is now required by the Post-Master General.*

### TO OUR SUBSCRIBERS.

Our subscribers will please—should any mistakes occur, either in name, post-office or otherwise—address the publisher, Mr. J. J. TOON, P. O. box No. 24, who will attend to them promptly. Should he fail to do so, then address the editors, and they will see to their complaints and have them rectified. Don't forget this. There is no harm to be done either party by having errors corrected, and we pledge ourselves to see that they shall be.

### CORRECTION.

Dr. Hodges, of Arkansas, writes to inform us that a mistake occurred in the February edition, in regard to the number of pills to be made from the formula of his "Sovereign Anti-Bilious Pill." The mass should be divided into sixty (60) pills. We trust our friends will make the correction in their copies. We will be pleased to hear from Dr. H. again.

### PLEASE REMEMBER.

The publisher desires to remind those of our subscribers who have not yet paid for THE COMPANION of last year, of the fact. Every subscriber knows whether he has paid or not. If you fall, friend, among the latter class, will you not send in your two-dollars at once? Don't forget this! Those of our subscribers, also, of this year, who have not yet paid, will please remember to do so *at once.*

Now friends, one and all, remember to send along your two dollars, without delay—if you have not already done so.

### APOLOGETIC.

We have been unable to notice, in this number, from various causes, the books, pamphlets, etc., sent us. We will attend to this in our next. In the meantime we hope our friends will bear with us.

### THE AMERICAN MEDICAL ASSOCIATION.

We have received from W. B. Atkinson, M. D., Permanent Secretary, notices in regard to the approaching meeting of the above-named body—its Twenty-third Annual Session.

From the large list of distinguished names on the different committees, the medical gentlemen of the country may expect a meeting of unusual scientific interest.

Arrangements of a most satisfactory character have been made with the Hotels and Boarding-houses of Philadelphia, by Dr. Atkinson, for the accommodation of all delegates. The railroads have, generally, agreed to transport delegates for one fare, but those who desire to avail themselves of this, must send to the Secretary their full names and the names of all the railroads over which they must travel in going to the meeting, with stamp for postage.

To our friends of Georgia and Alabama, who expect to pass through Atlanta, we call attention—don't forget the name—to the "Western and Atlantic, excursion tickets—one fare," which will afford them the most direct and convenient route from this point, via Knoxville, Tenn,, Lynchburg, Va., Washington, D. C., to Philadelphia.

For the benefit of those who expect to attend, we append the following:

HOTEL ARRANGEMENTS:

Continental, Chestnut and 9th, $4 a day,
Girard, Chestnut and 9th, $3 a day,
La Pierre, Broad below Chestnut, $3 a day,
Colonade, Chestnut and 15th, $3 a day,
St. Cloud, Arch below 8th, $3 a day,
St. Elmo, Arch above 2d, $2 50 a day,
American, Chestnut below 6th, $2 50 a day,
Merchants, 4th above Market, $2 50 a day,
St. Lawrence, Chestnut below 12th, $2 a day,
Alleghany, Market below 9th, $1 75 a day,
St. Charles, 3d below Arch, lodging only, 50 cents a day,
Miller's, 7th and Chestnut, lodging only. $1 50 a day,

Meals at Restaurant of Horticultural Hall, and Petry's, N. W. corner of Broad and Walnut, each 50 cents.

BOARDING HOUSES:

318 South Broad, $2 a day,
N. E. corner of Broad and Spruce, $1 50 a day, or $10 a week.
329 South Broad, $2 a day, or $10 a week.
1327 Spruce Street, $2 a day, or $12 a week,
225 South Broad, $2 50 a day, or $12 a week.

